# Degradation of Cellulose Derivatives in Laboratory, Man-Made, and Natural Environments

**DOI:** 10.1021/acs.biomac.2c00336

**Published:** 2022-06-28

**Authors:** Nejla B. Erdal, Minna Hakkarainen

**Affiliations:** KTH Royal Institute of Technology, FibRe − Centre for Lignocellulose-based Thermoplastics, Department of Fibre and Polymer Technology, Teknikringen 58, SE-100 44 Stockholm, Sweden

## Abstract

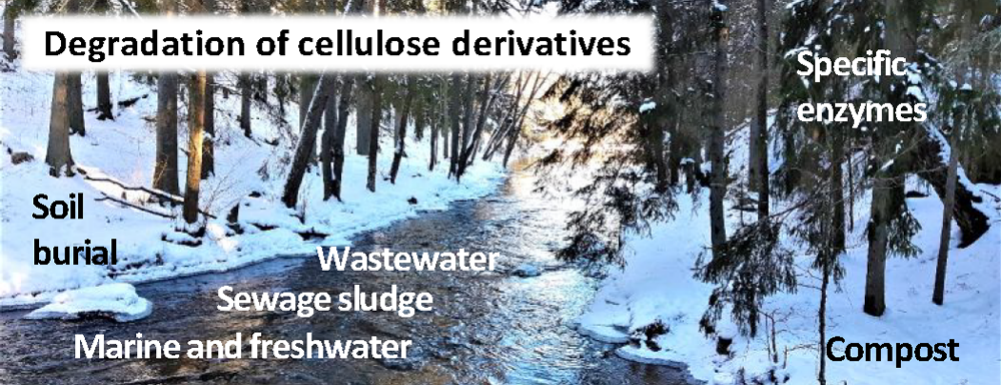

Biodegradable polymers complement recyclable materials in battling plastic waste because some products are difficult to recycle and some will end up in the environment either because of their application or due to wear of the products. Natural biopolymers, such as cellulose, are inherently biodegradable, but chemical modification typically required for the obtainment of thermoplastic properties, solubility, or other desired material properties can hinder or even prevent the biodegradation process. This Review summarizes current knowledge on the degradation of common cellulose derivatives in different laboratory, natural, and man-made environments. Depending on the environment, the degradation can be solely biodegradation or a combination of several processes, such as chemical and enzymatic hydrolysis, photodegradation, and oxidation. It is clear that the type of modification and especially the degree of substitution are important factors controlling the degradation process of cellulose derivatives in combination with the degradation environment. The big variation of conditions in different environments is also briefly considered as well as the importance of the proper testing environment, characterization of the degradation process, and confirmation of biodegradability. To ensure full sustainability of the new cellulose derivatives under development, the expected end-of-life scenario, whether material recycling or “biological” recycling, should be included as an important design parameter.

## 1 Introduction

In circular bioeconomy, materials need to be designed for specific and managed end-of-life scenarios that will prevent waste production and accumulation.^1^ Whether this will be organic recycling through biodegradation or mechanical or chemical recycling depends on the type of material and especially on the application where the product is used. When it comes to synthetic and biobased polymers including cellulose-derived materials, their design for biodegradability or compostability alone cannot solve the current waste problem.^2^ In most cases, material recycling is the most sustainable option to utilize the material value of the product that has come to the end of use phase. For some applications, a durable product with long service life is the most sustainable option, while other products, such as most packaging products, are aimed at short-term use. Some products have high risk or are even expected to end up in the environment or compost. Even when used correctly, the collection and recovery can be difficult or the products might be contaminated with organic matter, making recycling challenging.^3^ These are applications where biodegradability is a favorable property and can help to prevent waste from accumulating and polluting natural environments.^4^ Biodegradation is an attractive property, especially for many products in agriculture and forestry (e.g., mulch films, binding yarns, flocculant aids, control release carrier substances, seed coatings, and tree shelters), household and gardening (biowaste bags, wet wipes, sanitary items, packaging for dishwasher tabs, coffee capsules, microplastics in cosmetics, and tea bags), fishery products, and other products, such as geotextiles. The market volume for these applications in Europe alone was estimated to be 1 million tons per year.^5^ Many of these products are currently made of nondegradable plastics, and a change to (bio)degradable materials could bring environmental and practical benefits.

Biodegradation can be divided into aerobic and anaerobic processes, which produce water, carbon dioxide and/or methane, mineral salts, and the growth of biomass. The term biodegradation itself does not contain any information concerning the time scale, location, or degree of degradation required. It can mean ultimate mineralization or only structural changes due to biological activity. In the literature, the use of this term is also contradictory and can indicate everything from the mere growth of microorganisms on the surface of the materials to weight loss or complete mineralization of the material proved by release of CO_2_. Commonly, a contradiction exists between important material properties and processability versus retained biodegradability, especially when developing materials for applications such as food packaging, where good barrier properties are often required. The improvement of barrier properties generally also improves the resistance toward biodegradation.^6^ It is challenging to design a material that has good processability and material properties, is inert during the service life, and then rapidly and completely biodegrades to CO_2_ after disposal.

Understanding the influence of chemical modification on (bio)degradability of the material in different environments provides tools for designing materials for degradability in an expected end-of-life environment. At the same time, this understanding can also be utilized for the design of durable materials for long service-life, recyclability, and/or resistance against biodegradation and microbial attack. The expected end-of-life options for biodegradable plastics include industrial and home compost, soil, wastewater, freshwater, and seawater. The degradation rate of materials in different man-made or natural environments will depend on the combination of the material–environment, but it is generally fastest in industrial compost with controlled conditions, high temperature, humidity, and a high concentration of microorganisms, while in freshwater and saltwater, the average temperature and concentration of microorganisms are much lower (Table 1). A recent extensive data analysis of the literature results showed that average biodegradation levels reported for different biodegradable materials were 72% after 75 days in industrial compost, 47% after 155 days in a marine environment, and 40% after 159 days in soil.^7^ It should also be kept in mind that the degradation taking place in these environments is not only biodegradation.^8^ Degradation in natural and man-made environments is typically caused by a combination of abiotic and biotic mechanisms including biodegradation, chemical hydrolysis, photodegradation, oxidation, mechanical wear, and thermal degradation.^9^ The initial abiotic degradation can facilitate or even be a prerequisite for subsequent biodegradation. Polymers and biopolymers are too large to be taken up by the microorganisms, so they are often first degraded by abiotic mechanisms or by extracellular enzymes. Smaller compounds that are cleaved off can then be taken up by microorganisms to be further degraded by endoenzymes.^10^ Cellulose can also be directly degraded by cellulosomes, large extracellular enzyme complexes. Mechanical degradation can be caused, for example, by the action of wind and waves or meso- and microfaunal activities (e.g., earthworms).^11^

**Table 1:** Typical Conditions Found in Different Environments As Well As General Requirements Found in Standards Certifying Ultimate Degradability in Different Environments^5,12^

	temperature (°C)	pH	microbes (mL)	degradation standard requirements
industrial compost	50–60	6.5–8	10^9^	*t* = 58 °C
				>90% degradation in 180 days
home compost	25–70	6.5–8	<10^9^	*t* = 28 °C
				>90% degradation in 1 year
soil	<35	5.5–8	10^5^–10^9^	*t* = 25 °C
				>90% degradation in 2 years
sewage sludge	<37	5.5–8	10^6^–10^9^	
freshwater	0–25	6–9	10^3^–10^6^	*t* = 21 °C
				>90% degradation in 56 days
seawater	0–30	7.5–8.4	1–10^5^	*t* = 30 °C
				>90% degradation in 180 days

According to standard test methods, material is classified as inherently biodegradable if it degrades to >70% in a maximum of 10 days in aquatic aging tests measuring biological oxygen demand (BOD) or dissolved organic carbon (DOC). The 10 days are counted from the time when 10% of the material has degraded.^13^ To be certified as biodegradable in certain defined environments, >90% degradation as measured by oxygen demand or evolved CO_2_ is generally required (Table 1). 90% CO_2_ production has been selected as the limit instead of 100%, because some carbon can be incorporated into biomass or transformed into carbonic acid during the process.^14^ Several recent reviews summarize the used standards and their main characteristics.^15,16^ When one looks at the conditions defined in the standards that are used to certify materials as degradable in different environments, it is clear that the conditions in these standard tests are likely more favorable (e.g., temperature is typically kept at a maximum of what could be expected) than the conditions in real natural or man-made environments.^4^ The actual degradation in soil or marine water, for example, could thus take longer than the degradation time measured by these standard test methods.^17^ This is motivated by practical reasons to reach a balance between accuracy and efficient testing. Laboratory tests should however be complemented with more testing under real natural environments. At the end, it is impossible to fully simulate actual environmental conditions, which vary greatly even for the same “type” of degradation environment. As an example, soil burial can take place in different locations, seasons, and types of soil. At the same time, degradation in open natural environments can be promoted by factors like photooxidation caused by sunlight, chemical hydrolysis, and mechanical forces. The current knowledge on biodegradability of plastics in the open environment was summarized in the recent SAPEA report.^18^

In the literature, the degradation of polymers, biopolymers, and bioplastics is commonly measured by weight loss, molecular weight changes, and mechanical property loss. However, weight loss can also be caused by dissolution of low molecular weight compounds or additives, or it can be caused by fragmentation and not ultimate biodegradation of the polymer. The formed degradation products and released additives should be nontoxic and further biodegradable so that they will not accumulate in natural environments for any longer periods of time. Following material changes caused by degradation in different environments gives valuable scientific knowledge on the degradation mechanisms. However, the ultimate biodegradation under aerobic or anaerobic conditions should be proven by the release of CO_2_ or CO_2_ and CH_4_, or alternatively, biological oxygen demand can be used under aerobic conditions.

## 2 Cellulose and Common Cellulose Derivatives

Biopolymers like cellulose are inherently biodegradable in natural environments although the rate can vary largely depending on the type of environment and biopolymer. The chemical modification of cellulose is required for many material applications, and this may disturb the inherent biodegradability. Already, some of the earliest plastic materials were prepared by chemical modification of cellulose. In 1855, the first thermoplastic polymer, nitrocellulose or celluloid, was invented. The most important cellulose derivative in current commercial production is cellulose acetate (CA). The degradation of cellulosic biomass plays an important role in the carbon cycle within the biosphere. Cellulose, similar to starch, consists of glucose monomers. In the cellulose chain, they are mainly linked together by β-glycosidic linkages, compared to α-glycosidic linkages in starch. Cellulose chains form highly crystalline fiber structures and participate in extensive hydrogen bond networks. This makes cellulose a relatively recalcitrant material that is harder to decompose than other polysaccharides.^19^ In addition to chemical structure, morphology and degree of crystallinity are important for biodegradability of a polymer and may vary with the origin of the material, extraction processes, chemical modifications, and processing, among others.^20^ In nature, cellulose is naturally biodegraded by various microorganisms. Degradation is carried out by enzymes called cellulases secreted by cellulolytic bacteria and fungi. These can be divided into endoglucanases, which are capable of hydrolyzing the β-1,4-glycosidic linkages present in amorphous cellulose and cellobiohydrolyses that can react with the end groups of cellulose.^21^ Lytic polysaccharide monooxygenases can also contribute to oxidative cleavage of glycosidic bonds. Complete biodegradation of cellulose ultimately results in carbon dioxide and water under aerobic conditions and carbon dioxide, methane, and water under anaerobic conditions as well as biomass.^22^

**Figure 1:**
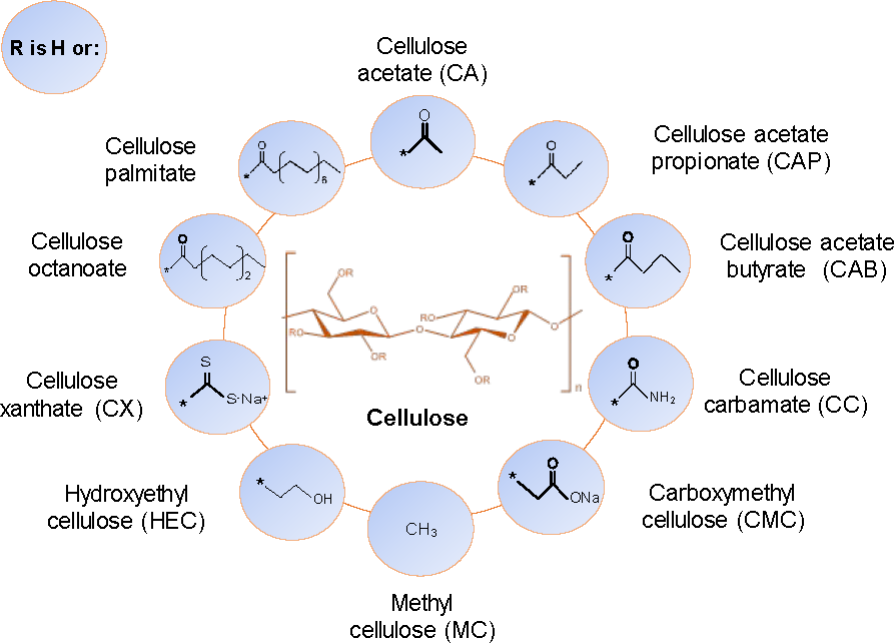
Chemical structure of cellulose and some of its common derivatives where the R can be a hydrogen (H) atom or one of the groups presented in the figure.

Cellulose is insoluble in water and in most organic solvents, and it also decomposes at elevated temperatures before melting. This is due to the strong hydrogen bonding network and highly ordered crystalline structure. The many functional groups of the cellulose structure have been utilized for chemical modifications to introduce, e.g., thermoplastic properties or solubility in different solvents.^23^ The substitution of the hydroxyl groups on the cellulose backbone with different types of substituents yields a wide range of cellulose derivatives with different properties. Chemical structures of common cellulose derivatives are presented in Figure 1. The type of chemical modification and degree of substitution (DS), i.e., how many of the hydroxyl groups on each glucose unit have reacted, has a large influence on material properties, processability, and biodegradability.^24^

Among the different cellulose derivatives, CA, has been extensively studied. CA can be found in a variety of consumer products such as plastic films, textiles, and cigarette filter tows. It is obtained through acetylation of some of the hydroxyl groups of cellulose. The most common DS of thermoplastic CA products is around 2.5, which yields good solubility in common solvents and melt processability.^25^ Early research revealed that the DS is an important factor when determining the susceptibility of CA and other cellulose derivatives to biodegradation.^26,27^ Other commercially important cellulose esters are cellulose acetate propionate (CAP) and cellulose acetate butyrate (CAB). Long-chain cellulose esters are not currently produced commercially because of the high price, but they could be potential candidates for various applications such as films and composites.^28−30^ They have also been evaluated for drug delivery and tissue engineering purposes.^31^ Cellophane (CP), regenerated cellulose, is produced from cellulose by harsh chemical processing via cellulose xanthate (CX), and it is mainly used in the form of films, for example, in food packaging.^32^ Cellulose carbamate (CC) is a carbamic acid ester derivative of cellulose. It is produced by reacting cellulose with urea. The solubility of CC in alkaline solutions has gained attention for fiber regeneration from wood pulp as an alternative to the viscose process. Its use as a textile fiber but also in films, membranes, and foams has been reported. An important advantage is the relatively high stability of CC at room temperature, allowing long storage.^33^

Cellulose ethers are a major class of commercially important, often water-soluble, cellulose derivatives. One of the simplest cellulose ethers is methyl cellulose (MC).^34^ MC is used as a thickener in the food industry, as an admixture for concrete in civil construction, and in controlled drug delivery applications in the pharmaceutical industry.^35,36^ Another cellulose ether derivative is hydroxyethyl cellulose (HEC), which is used in a wide range of industries including food, oil recovery, cosmetics, pharmaceuticals, adhesives, printing, textile, construction, paper, and agriculture.^37^ Carboxymethyl cellulose (CMC) is also a water-soluble ether derivative in which part of the hydroxyl groups of cellulose have been replaced by carboxymethyl groups. CMC with the DS ranging from 0.4 to 1.3 has been widely used as a detergent and food additive.^31^ Nanocelluloses, including cellulose nanofibrils (CNF) and cellulose nanocrystals (CNCs) prepared from wood and other plant celluloses, have rapidly emerged as important cellulose derivatives with a wide range of applications in composites, packaging, coatings, biomedical, construction, and electronics to name a few^38−41^ Due to the high hydrophilicity of these materials, chemical modification is typically needed for the preparation of cellulose nanocomposites with more hydrophobic plastic materials or for the reduction of the hydrophilicity of the products.^42^

The chemical (e.g., DS and type of substituent) and physical structure of cellulose derivatives will majorly affect the susceptibility to biodegradation, which will be further influenced by “environmental factors” in the given location.^9^ The environmental factors are difficult to control, and the degradation rate of materials is in general difficult to predict in real natural environments with strong influences from, e.g., location, season, temperature, UV light exposure, salinity, and humidity.^43,44^ Degradation of plastics and bioplastic including chemically modified biopolymers can be investigated in real environments or more commonly by different laboratory tests investigating the effect of single or multiple parameters or by trying to simulate or accelerate conditions during, e.g., composting or aging in natural environments. The following sections will summarize the current knowledge on the degradation of cellulose derivatives in different degradation environments to provide insights on structure–environment–(bio)degradability relationships.

## 3 Degradation by Specific Enzymes

Laboratory testing of enzymatic degradation is a relatively simple degradation test. Relevant control experiments should still be included to confirm the enzyme-catalyzed process over chemical hydrolysis. This test can also be more favorable compared to real environments as the temperature and the type and concentration of enzymes can be optimized. In addition to the chemical structure and composition, the physical structure will influence the enzymatic degradation process.^45^ In a study from 1957, it was elaborated that, while cellulose was 100% degraded by enzymes (cellulase and β-glucosidase), many of its ester and ether derivatives did not degrade to the same extent. Among the three investigated esters, cellulose sulfate (DS of 0.40), CA (DS of 0.76), and cellulose acetate phthalate (DS of 2.2), the enzymatic biodegradation was the highest for cellulose sulfate. As it also had the lowest DS, it is difficult to conclude if the chemical structure or DS was the main determining factor. Among the ether derivatives sulfethylcellulose, CMC, HEC, carboxymethyl hydroxyethyl cellulose (CMHEC), and MC, the enzymatic degradation rates were slightly lower than those of the ester derivatives when comparing materials with similar DS values. The lowest enzymatic degradation was observed for CMC and MC with high DSs. The author speculated that the DS as well as the type and location of the substituent could influence the degradability.^26^ This was later confirmed by a very detailed study of randomly and regioselectively substituted CA with the DS varying from 0.4 to 2.1, showing that the location of the substituent and type of cellulase had a large influence on whether hydrolysis took place or not.^46^ A detailed description of enzymatic degradation of CA has also been presented.^25^

The enzymatic degradation of chemically modified cellulose materials seems to require a cocktail of enzymes. As an example, CA with a high degree of acetylation requires a two-step process: first, the degree the acetylation needs to be reduced by chemical hydrolysis (deacetylation) or by enzyme-catalyzed hydrolysis with acetyl esterases. Some studies indicate that already for materials with a DS of 0.9–1.8 deacetylation is required, while others showed that some enzymatic degradation is still possible within this DS range.^47^ After reduction of the degree of substitution, cellulases can hydrolyze the β-1,4-linkages in the cellulose backbone.^48^

Enzymatic hydrolysis of different cellulose-based materials by a mixture of cellulases led to the following degrees of hydrolysis after 6 h at 50 °C: cellophane 78%, cotton fabric 31%, unbleached kraft paper 43%, sausage casing 82%, aminated bleached kraft pulp 0.2%, and cellulose acetate 0%.^45^ This further supports that a combination of cellulases with enzymes that are capable of deacetylation is required to achieve significant enzymatic degradation of chemically modified cellulose materials.^49^ However, even the enzymatic deacetylation rate can be inhibited by a high DS. A recent study evaluated several different enzyme systems for deacetylation of CA with different DSs. There were in most cases only minor differences in the deacetylation rate of DS 0.9 and 1.4 materials, while the tested esterases were not able to deacetylate CA with a DS > 1.8. The DS, thus, has crucial influence even on the enzymatic deacetylation.^24^

The enzymatic degradation rate of cellulose ethers also highly depends on the DS of the samples, which aligns with what has been shown for cellulose esters. Enzymatic degradation of 11 cellulose ethers, including six CMCs (DS of 0.41, 0.79, 0.89, 0.97, 1.30, and 2.45), three HECs (DS of 1.1, 1.2, and 1.6), one MC (DS of 1.8), and one hydroxypropylcellulose (HPC) (DS of 2.1), by a commercial cellulolytic enzyme complex indicated that all cellulose ethers with DS < 2 were significantly degraded. For the materials with DS > 2, the enzymatic hydrolysis rate significantly slowed down. In the case of CMC, the enzymatic hydrolysis rate significantly decreased for the samples with DS > 1, while MC with smallest substituent degraded more readily than the other cellulose ethers.^50^

**Figure 2:**
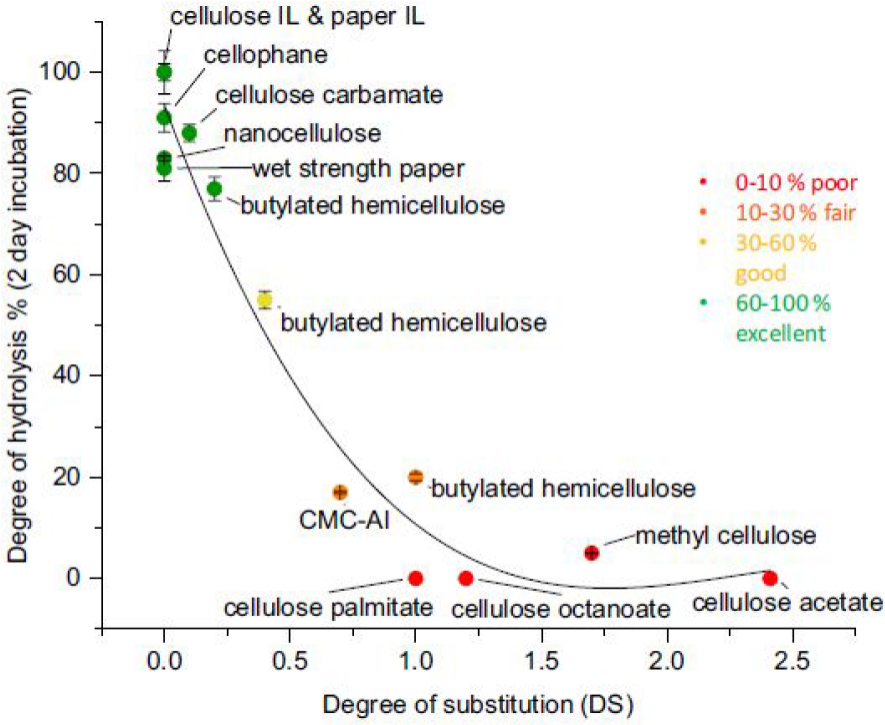
Enzymatic hydrolysis of common cellulose derivatives where the degree of hydrolysis (%) is shown as the function of the degree of substitution (DS) after a 2 day incubation. Reprinted by permission from ref (51). Springer Nature, *Journal of Polymers and the Environment*, Enzymatic Degradation and Pilot-Scale Composting of Cellulose-Based Films with Different Chemical Structures, Leppänen, I.; Vikman, M.; Harlin, A.; Orelma, H. Copyright 2020 http://creativecommons.org/licenses/by/4.0/ (No changes were made to the copyrighted material).

Recently, an interesting comparison was performed on enzymatic degradability of 14 different cellulose materials, including, e.g., regenerated cellulose, nanocellulose, CMC, MC, CA, CC, CP, cellulose palmitate, cellulose octanoate, and wet strength paper (Figure 2). An enzyme cocktail consisting of cellulase, mannase, xylanase, and β-glucosidase was utilized. The effect from both the DS and the type of substituent on the enzymatic hydrolysis rate was clear. The nonsubstituted cellulose materials were 80–100% hydrolyzed during the 2 day hydrolysis period, while the hydrolysis rate decreased to almost 0% for the materials with longer substituents or a high DS. Butylated hemicellulose with a DS of 0.2, 0.4, and 1.0 further confirmed the decreased hydrolysis rate going from the material with the lowest DS toward the material with a higher DS.^51^ With the help of quartz crystal microbalance, it was shown that a mixture of endoglucanases and cellobiohydrolases can have synergy effects to swell and degrade cellulose films.^52^ In addition to chemical functionalization, blending influences the accessibility of the cellulose chains to enzymatic degradation. As an example, the modification of easily enzymatically hydrolyzable bacterial cellulose with lignin nanoparticles significantly retarded the enzymatic degradation rate, and the type of lignin further influenced the observed hydrolysis rate.^53^

The susceptibility of material to enzymatic or biodegradation can often be enhanced by UV irradiation (as simulated sunlight). A combination of deacetylating enzymes (lipase or esterase) and cellulase did not significantly promote the degradation of CA with DS = 2.4. However, when the same CA was UV irradiated before suspension in sterilized buffer or cellulase solution, 23% and 60% weight loss took place, respectively.^54^ H_2_O_2_ producing enzymes, cellobiohydrolase and cellobiosedehydrogenase, have also been adsorbed on cellulose films aimed at antibacterial surfaces. However, as other studies have demonstrated that UV irradiation in combination with H_2_O_2_ can trigger rapid self-destruction of cellulose materials, this approach could possibly also be utilized for the preparation of self-degradable cellulose films.^55^

## 4 Degradation in Wastewater and Sewage Sludge

**Figure 3:**
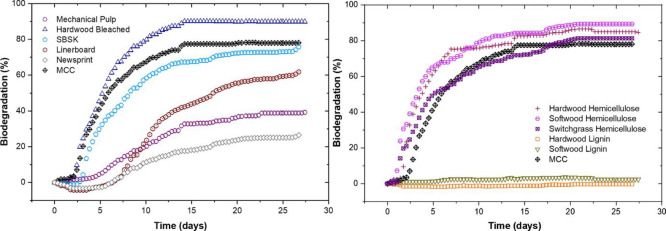
Extent and rate of biodegradation of different lignocellulose materials. Reprinted by permission from ref (57). Springer Nature, *Cellulose*, Effect of lignocellulosic fiber composition on the aquatic biodegradation of wood pulps and the isolated cellulose, hemicellulose and lignin components: kinetic modeling of the biodegradation process, Kwon, S.; Zambrano, M.C.; Pawlak, J. J.; Venditti, R.A. Copyright 2021.

Cellulose derived products have a high risk of ending up in wastewater and thereafter in sewage sludge. It is therefore important to know their behavior and potential biodegradability in these environments. Furthermore, the interest in using wood fibers for the fabrication of single-use products has increased rapidly in the last years. Wood pulp composition including lignin content varies depending on the type of wood and pulping process and can have a large influence on the degradation process. The biodegradation of lignocellulosic fibers has been studied in many works and will not be discussed in detail here. For the degradation of lignocellulosic fibers, hydrolytic and ligninolytic extracellular enzymes are generally required. Hydrolyses can hydrolyze cellulose and hemicellulose, while lignin degrades more slowly and by a nonhydrolytic process.^56^ To illustrate this, aquatic biodegradation of wood pulps with different compositions was investigated according to ISO 14851 with inoculum from a wastewater plant. The included materials were mechanical pulp, bleached hardwood, southern bleach softwood kraft, linerboard, and newspaper with a total lignin content of 31.2%, 1.2%, 1.9%, 17.5%, and 18.9% and a corresponding carbohydrate content of 67.7%, 99.8%, 99.3%, 79.9%, and 79.4%, respectively. Figure 3 shows the biodegradation rate of these materials. MCC was included as a positive control. It is clear that the materials with high carbohydrate content and low lignin content were more rapidly and completely biodegraded, while the materials with a higher lignin content degraded to a much lower degree. It can also be pointed out that cellulose crystallinity was similar for all the above materials (57–66%) with the exception of MCC that had a higher crystallinity of 85%. This further supports that the differences in the biodegradation rate were mainly deduced from the composition of the materials. When the biodegradation of softwood and hardwood hemicelluloses and lignins was tested separately, fast and nearly complete biodegradation of both hemicelluloses and basically no biodegradation of the corresponding lignin were illustrated (Figure 3). This could, possibly in combination with the generally slow biodegradation rate of lignin, indicate the absence of lignin degrading microorganisms in the inoculum.^57^

Cellulose, kraft paper, sausage casing, and cotton fabric were rapidly biodegraded to more than 60% in a modified Sturm test within 10 days (sewage sludge at 25 °C). After 22 days, the evolved CO_2_ was 79%, 77%, 70%, and 74%, respectively, of the theoretical value. In comparison, the cellulose derivatives, CA, and aminated cellulose with high a DS of >2.5 were not biodegradable in this test, showing less than 10% CO_2_ generation. An interesting finding was also that the cellulose fabric containing highly crystalline cellulose was readily biodegraded in the modified Sturm test by sludge microorganisms, while at the same time it was not hydrolyzed to a large extent by isolated enzymes.^20^

**Figure 4:**
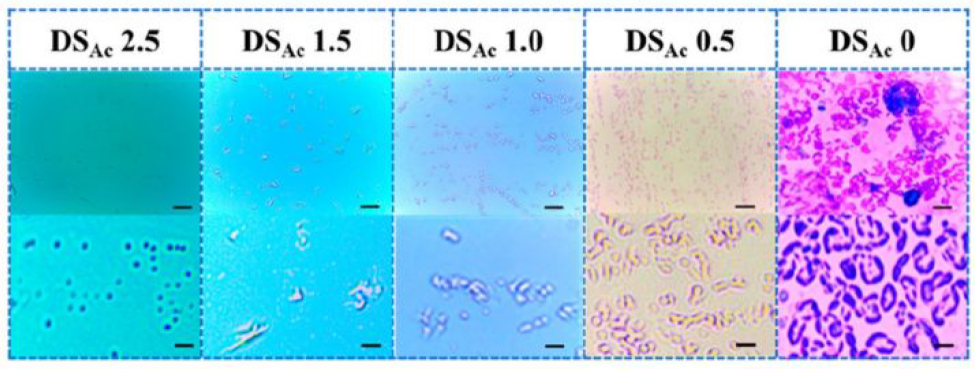
Influence of the DS on the ability of *B. subtilis* to adhere on the surface of the CA films. Reprinted from ref (58). Copyright 2022 American Chemical Society.

Aerobic biodegradation of CA with different DSs proceeded at a slower rate in an open wastewater treatment system compared to a closed batch system with enrichment culture from activated sludge. It took 27 days for CA with a DS of 1.7 to degrade to >70%, while it took 10 weeks to reach a weight loss of >10% for CA with a DS of 2.5. Basically, no changes were observed for DS 2.95 material. In the closed system, >90% of DS 1.7 samples degraded within 4–5 days, while significant degradation (67% weight loss) was observed after 11 days for DS 2.5 samples. Even here, the DS 2.95 samples remained basically unaffected after 28 days.^27^ A detailed study of CA deacetylated from a DS of 2.5 to 1.5, 1.0, 0.5, and 0 illustrated the dramatic influence of deacetylation and increased hydrophilicity on the ability of common soil bacteria *Bacillus subtilis* to adhere to the surface of CA films (Figure 4). As the DS decreased, there was a development from a few germinated spores to a large number of fully developed *B. subtilis* on the surface of the films.^58^

**Figure 5:**
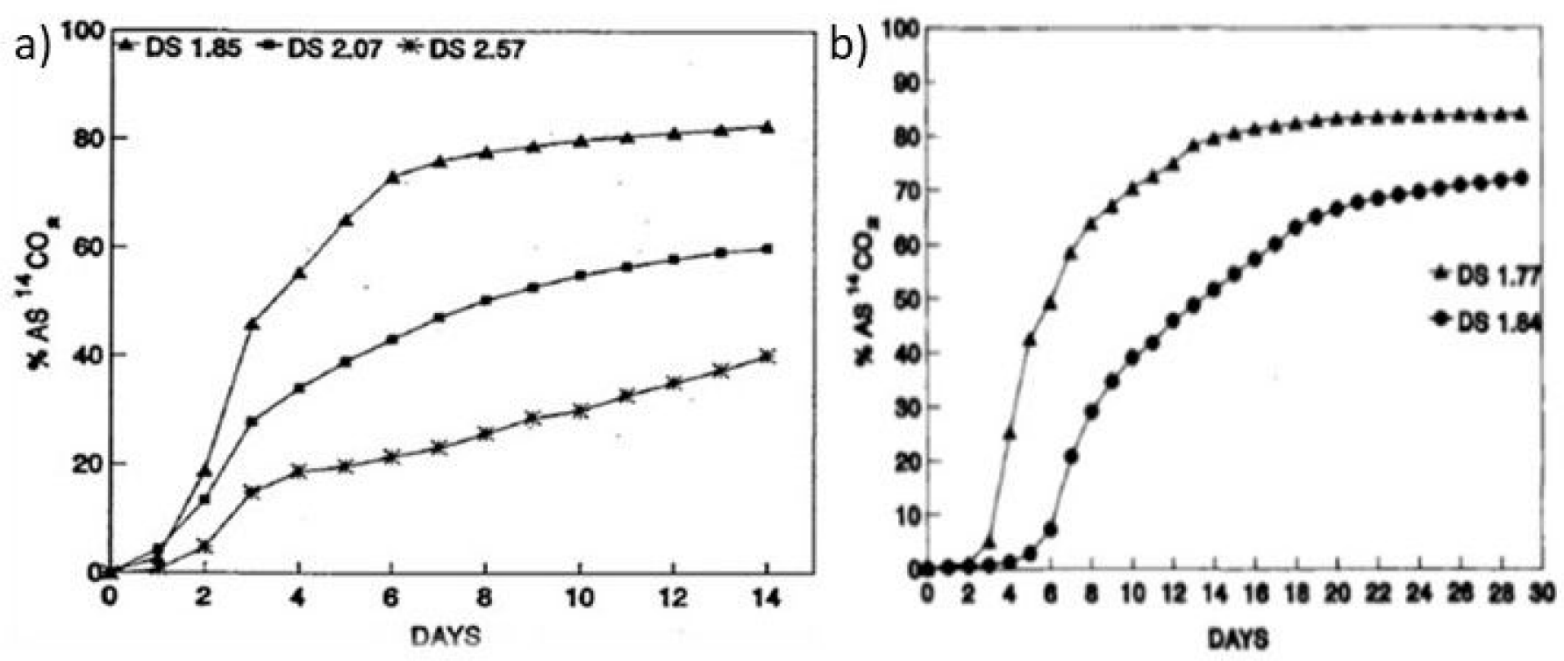
Biodegradation of (a) cellulose acetate with a DS of 1.85, 2.07, and 2.57 during 14 days and (b) cellulose propionate with a DS of 1.77 and 1.84 during 30 days. The degree of biodegradation was calculated from the release of ^14^CO_2_. Reprinted with permission from ref (59). Copyright 1993, John Wiley and Sons.

The utilization of radiolabeled monomers or polymers is highly interesting for the detailed mapping of a degradation process. The aerobic biodegradation of radiolabeled CA and cellulose propionate has been investigated in a mixed microbial culture derived from activated sludge. This study very effectively demonstrated not only the effect of the DS on CA degradation but also the effect of increasing the ester length from acetate to propionate. This change seems small but had a crucial effect on the biodegradation rate; see Figure 5 (observe the different time scales for a and b). The radiolabeled cellulose acetate with a DS of 1.85, 2.07, and 2.57 biodegraded to approximately 80%, 60%, and 40%, respectively, after only 14 days. A rather dramatic effect was observed when the acetate group was changed to slightly larger propionate. Cellulose propionate with a DS of 1.84 still degraded to approximately 50% after 14 days, and the degree of biodegradation increased to 72% after 29 days. However, for cellulose propionate with a DS of 2.11 and 2.44, basically no biodegradation (max 1.1%) took place during a 30 day period,^59^ illustrating again the significant effect of both the DS and the size of the ester group.

Biodegradability of water-soluble polymers, such as many cellulose ethers, is of high interest because of the high risk of ending up in wastewater and natural water systems. The aerobic biodegradation testing of cellulose ethers has led to a general conclusion that they are biodegradable if DS < 1.^60^ Biodegradation of water-soluble CMC with a DS of 0.7 was investigated in a prolonged closed bottle test and semicontinuous flow activated sludge (SCAS) test that simulated a sewage treatment plant. The incubation of CMC in the closed bottle test yielded 25% biodegradation after 28 days, followed by a slower biodegradation rate on prolonged testing reaching 58% after 110 days. In the SCAS test, prior to entering the bioreactor, CMC was added to raw sewage and therefore already partly biodegraded by the microorganisms. In this test, CMC was completely degraded with >90% degradation. Moreover, an aquatic toxicity test was performed on the degradation intermediates, and they were shown to be nontoxic.^61^ As expected, CMC with a DS of 0.44 was more readily degraded than CMC with a DS of 0.75, which in turn degraded faster than PVA. The biodegradation was performed during 8 weeks in an air-saturated aquatic biochemical oxygen demand (BOD) test with yeast extract and supernatant liquid from settled domestic wastewater.^61^ The fungal strain *Cheatomium globosum* (*C. globosum*) effectively degraded CMC in carpentry waste. However, the presence of zinc and iron inhibited the growth of *C. globosum*. This indicates that high amounts of iron and zinc in the soil could inhibit the cellulolytic fungi from degrading cellulose and lead to accumulation. These metals together with chromium and nickel are also often present in industrial effluents.^62^

**Figure 6:**
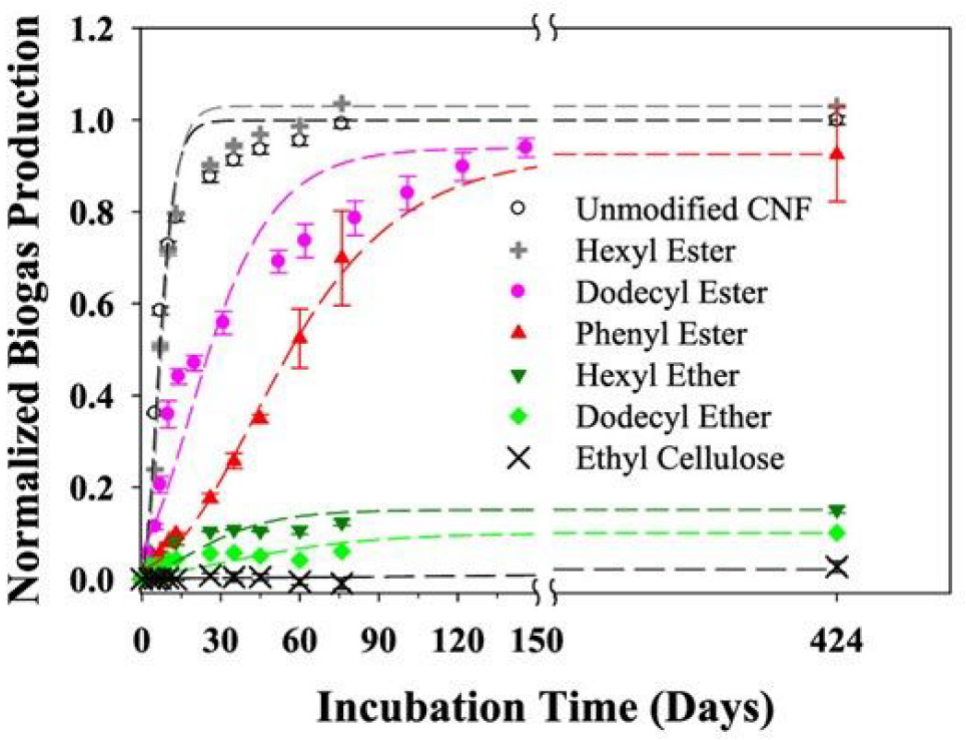
Normalized biogas production during anaerobic degradation of unmodified and modified CNF. Reprinted from ref (64). Copyright 2021 American Chemical Society.

It is important to confirm the potential environmental impacts of emerging commercial products like nanocellulose. The biodegradation of nanocrystalline cellulose (NCC) was compared with MCC by utilizing two anaerobic cellulose-degrading microbial consortia obtained from an anaerobic digester and wetland inocula. The biodegradation was assessed through the liberated glucose units during 11 weeks. Both materials readily biodegraded showing that the nanosize and high crystallinity did not inhibit the biodegradation rate. On the contrary, the rate was even slightly faster for NCC compared with MCC. This could be due to the larger surface area, although it was not further discussed by the authors.^63^ Chemical functionalization of nanocellulose products will also have an influence on the biodegradability (Figure 6). During the anaerobic biodegradation experiment, unmodified cellulose nanofibrils (CNFs) and nanocrystals (CNCs) degraded rapidly and were completely mineralized within 60 days. The degradation of hexyl-esterified CNF with a low DS proceeded almost as rapidly as the nonmodified CNF. Dodecyl and phenyl-esterified materials and TEMPO-oxidized CNF with carboxylate groups biodegraded at a clearly slower rate, but they were also completely mineralized after 424 days. On the contrary, almost no mineralization was observed on the etherified CNFs during 424 days. Similar trends were observed during degradation of the same CNF materials during aerobic biodegradation with inoculum from aerobic wastewater.^64^

**Figure 7:**
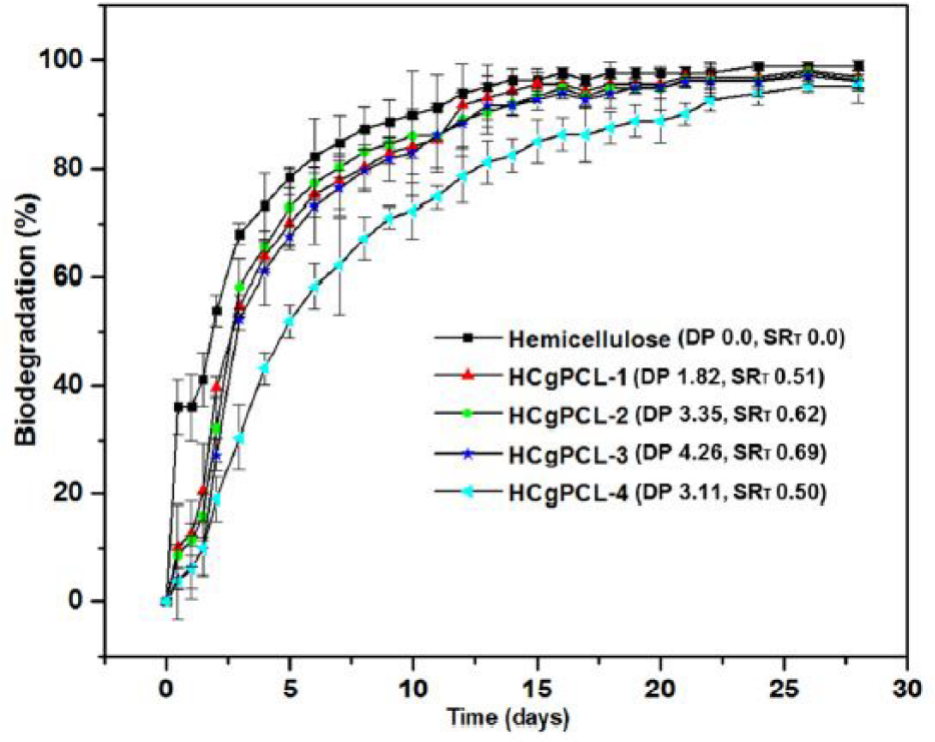
Aerobic biodegradability of hemicellulose and PCL-grafted hemicellulose using the standard ISO 14851 method. Reprinted from ref (65). *Materials & Design*, *153*, Farhat, W.; Venditti, R.; Ayoub, A.; Prochazka, F.; Fernández-de-Alba, C.; Mignard, N.; Taha, M.; Becquart, F. Toward thermoplastic hemicellulose: Chemistry and characteristics of poly(ε-caprolactone) grafting onto hemicellulose backbones, 298–307. Copyright 2018, with permission from Elsevier.

A possible route to the retainment of biodegradability after chemical modification could be grafting with readily biodegradable polymers instead of short ethers and esters. In addition to the biodegradability of the graft itself, this approach could reduce the DS required for reaching the targeted properties, leaving cellulose chains more susceptible to biodegradation. This was evaluated for hemicellulose, which was subjected to ring-opening graft polymerization of ε-caprolactone (CL). The average degree of polymerization for PCL was in the range of 1.82–4.26, and the hemicellulose substitution rates were between 50% and 69%. The biodegradation was studied using the BOD method with microorganisms under aerobic conditions in closed respirometer bottles. All of the PCL-grafted xylan materials still exhibited high ultimate biodegradability ranging between 95% and 100% after 28 days; see Figure 7. Approximately the same degree of biodegradation was reached for nonmodified and modified hemicellulose although the initial biodegradation rate was slightly slower for the grafted materials.^65^

## 5 Degradation in Marine Water and Freshwater

Degradation in marine and other open aquatic environments can proceed through, e.g., hydrolysis, photodegradation, biodegradation, thermo-oxidation, and mechanical degradation. The mechanism and rate will be highly influenced by the type of the material and type of the aquatic environment.^66^ Different freshwater environments include rivers, lakes, and wetlands as well as water columns and sediments. There are no widely utilized international standards to specifically confirm the biodegradation in natural unmanaged freshwaters, while existing standards for marine environments resemble those for testing biodegradation in wastewater.^67^ The testing temperatures in the standards vary between 13 and 30 °C; the maximal duration is between 3 and 24 months, and the CFUs are between 10^3^ and 10^6^ mL^–1^. The inoculum for laboratory experiments is typically obtained from sediments or seawater. Alternatively, selected strains can be utilized. The medium can be synthetic or real seawater. The average surface temperature of the ocean is 17 °C, which decreases to 0–4 °C lower down in the ocean. Many cellulases are more active under acidic environments, while natural waters generally are weakly alkaline. For example, seawater is has pH of 8–8.5, and the oxygen level and concentration of bacteria (10^5^–10^7^ per mL) are much lower compared to those in soil and compost.^12^ Generally, the degradation in natural aquatic environments thus proceeds significantly slower compared to the degradation in, for example, compost. This rate is expected to additionally decrease once the material sinks to the seafloor.

**Figure 8:**
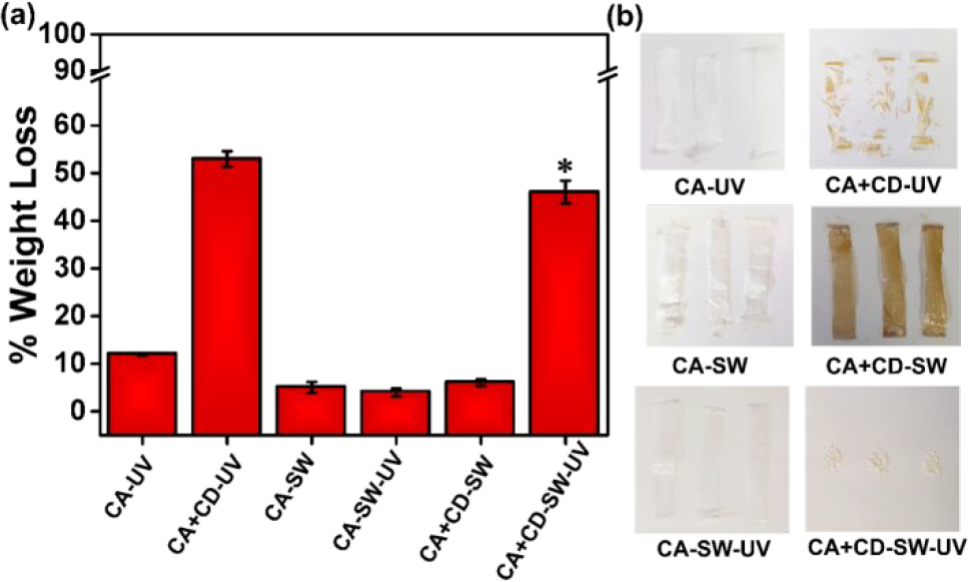
Weight loss of (a) CA and CD modified CA+CD films with and without UVA irradiation in air or in simulated seawater during 30 days. For the sample marked *, the real weight loss was likely even larger due to some salt deposition on the samples. (b) Images of the aged samples showing basically unaffected films with the exception of CD modified and UVA irradiated samples that had totally fragmented in agreement with the large weight loss. Reprinted from ref (71). Copyright 2021 American Chemical Society https://creativecommons.org/licenses/by/4.0/ (No changes were made to the copyrighted material).

Cigarette filters made of CA are one of the most common littered items in the world, including in coastal regions and oceans.^68^ The weight loss of CA cigarette filters was approximately 15% after a one year exposure in a laboratory artificial seawater microcosm kept at 25 °C and irradiated with UVB lamps.^69^ This can be compared to approximately 5% weight losses observed during a one year laboratory aging of CA in different aquatic environments including lake water, seawater, and artificial seawater at 25 °C without UVA irradiation. The main weight loss was observed during the first months and deduced mainly to deacetylation, which was confirmed by quantitative HPLC analysis. Some minor decrease of molecular weight also took place. When the temperature was increased to 60 °C, i.e., much above what is expected under natural conditions, there was only a minor accelerating effect, approximately doubling the weight loss. Although the degradation was not significant, the degree of acetylation decreased to below 2 during the first 1–3 months, which on the basis of previous studies could be enough to allow subsequent biodegradation to take place.^70^ Another related study illustrated that the modification of CA with carbon dots (CDs) produced by carbonization and oxidation of cellulose could significantly catalyze the deacetylation of CA under UVA radiation simulating sunlight. After 30 days of aging in artificial seawater, the weight losses of UVA irradiated CA and CD modified CA were 4 and 43 weight %, respectively, showing that CDs functioned as effective photocatalytic triggers (Figure 8). The degradation was shown by ^1^H NMR and SEC to be due to deacetylation and chain cleavage. This triggering effect is likely connected to the confirmed formation of H_2_O_2_ and subsequent radical formation when the carbon dots were UV irradiated in seawater or air. In accordance, the addition of H_2_O_2_ to the artificial seawater led to rapid degradation of CA on UVA irradiation, leading to a similar effect as the addition of CDs.^71^ In another study, polyphosphate incapsulated TiO_2_ photocatalyst was incorporated into CA films with the aim of inducing degradation if the material would end up in seawater. This seawater activated catalyst only led to collapse of the CA films when exposed to seawater and UV irradiation. Similar to the CD triggered degradation photo-oxidation, the catalytic action of TiO_2_ and benzophenone derivatives led to combined deacetylation and chain cleavage.^72,73^

**Figure 9:**
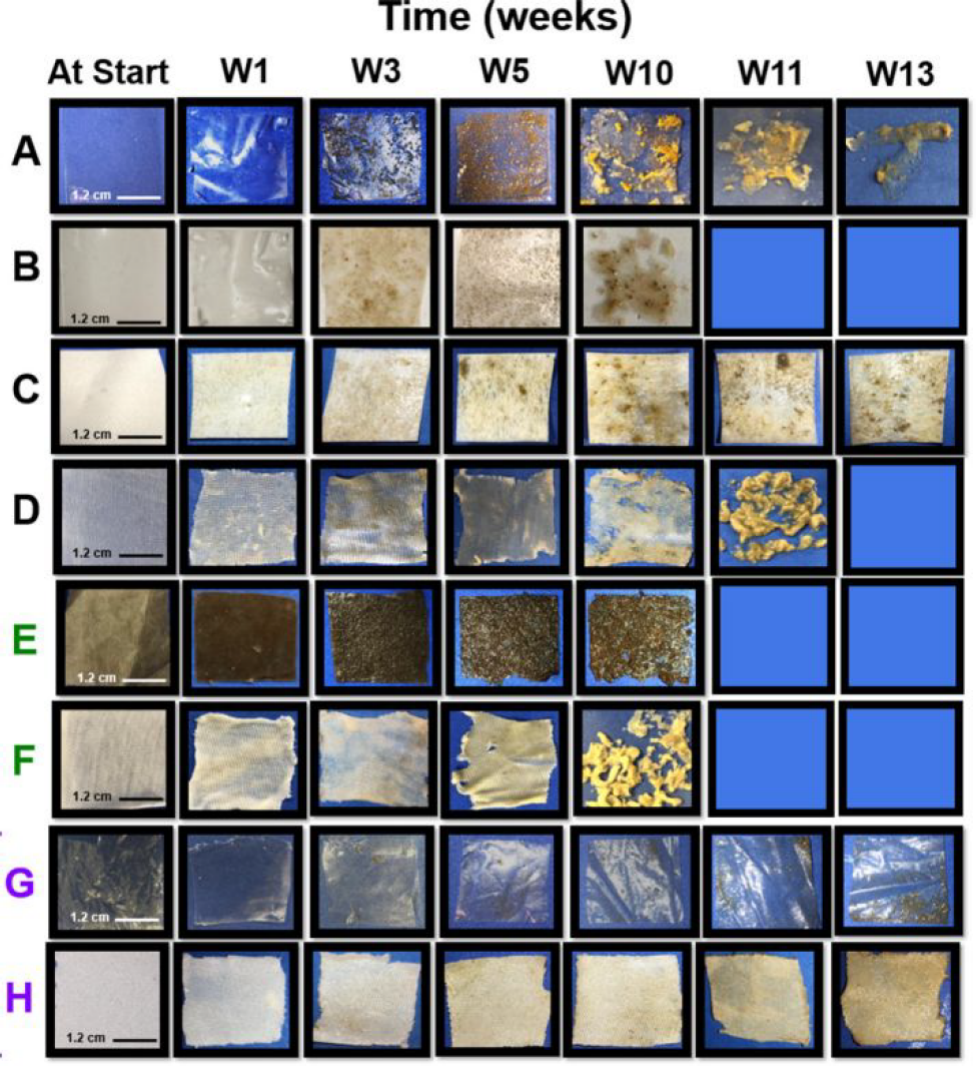
Images of (A) CA films, (B) plasticized CA films, (C) CA foams, (D) CA fabric, (E) kraft paper, (F) cotton fabric, (G) LDPE film and (H) PET fabrics after 0–13 weeks of incubation in a continuous flow seawater mesocosm. Reprinted from ref (74). Copyright 2022 American Chemical Society https://creativecommons.org/licenses/by-nc-nd/4.0/ (No changes were made to the copyrighted material).

The persistence of several cellulose acetate products (i.e., films, foams, and fabrics) in the ocean was evaluated by subjecting the products to a seawater mesocosm in continuous flow. All the tested CA products were shown to disintegrate within a maximum of three months. This rapid disintegration was deduced to the observed activity of esterases and cellulases, showing the presence of suitable microbial communities in marine water. The disintegration of CA took place with a similar rate to the positive controls (cellulose film and cotton fabric), while the negative controls (LDPE and PET) did not show any sign of degradation (Figure 9). This gives a positive indication of potential degradability of CA in a marine environment within a relatively short time frame.^74^ The seawater in this experiment was tempered to 20 °C, which is higher than the average during real marine exposure, and the complete biodegradation should be further proved by CO_2_ measurements. Another study investigated the degradation of CAB under real marine conditions in the Baltic Sea during a period of 25 weeks. The weight loss of CAB during the whole incubation time was 1.9%, which is very low. The substituents and steric hindrance were mentioned as factors preventing the microorganisms from degrading the material. In an attempt to improve the degradation rate, organic–inorganic cellulose acetate butyrate hybrids were synthesized using tetraethyl orthosilicate (TEOS) and triethyl phthalate (DEP). After 25 weeks of incubation in seawater, organic–inorganic hybrid CAB with 6% TEOS and 25% DEP or 12% TEOS and 25% DEP lost 17% and 18% of their weight, respectively, while CAB modified with only DEP lost 12%.^75^ The incorporation of CNF in polylactide films increased the otherwise slow degradation of polylactide in the simulated marine environment at 25 °C with periodic fluorescent illumination.^76^

916 seawater samples illustrated that oceanic microfibers in a large part consisted of cellulosic fibers.^77^ The degradation of cellulosic fibers in marine and other aquatic environments is thus of great interest. River water, brackish water, and seawater were collected, and the extent of biodegradation of cellulosic fibers was followed by measuring the weight loss and BOD during 30 days at 20 °C. The fiber samples included ramie, mercerized ramie, and regenerated cellulose. All the fibers lost 50–90% of their weight during 30 days depending on the aquatic environment. The biodegradation rate was more dependent on the type of water than on the type of cellulose fiber, reaching 40%, 20–30%, and 2–10% in river water, brackish water, and seawater. On the contrary, when the same fibers were evaluated for enzymatic degradability by a mixture of cellulase and β-glucosidase, large differences were observed depending on the type of fiber and the rate decreased in the order regenerated cellulose (53% degradation), mercerized ramie (18% degradation), and ramie (8% degradation). This was explained by a lower crystallinity and larger surface area in the case of regenerated cellulose.^78^

**Figure 10:**
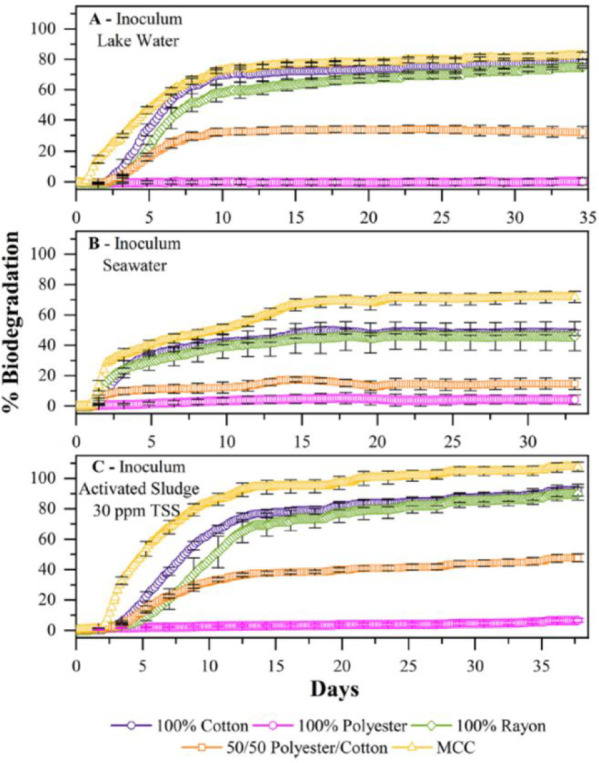
Biodegradation rate of cotton, polyester, rayon, 50/50 polyester/cotton, and MCC as measured by the oxygen uptake. The inoculums originated from lake water, seawater, and activated sludge. Reprinted from ref (79). *Marine Pollution Bulletin*, *151*, Zambrano, M. C.; Pawlak, J. J.; Daystar, J.; Ankeny, M.; Goller, C. C.; Venditti, R. A. Aerobic biodegradation in freshwater and marine environments of textile microfibers generated in clothes laundering: Effects of cellulose and polyester-based microfibers on the microbiome, 110826. Copyright 2020, with permission from Elsevier.

The aerobic biodegradation of common textile fibers was also evaluated using microbes from lake water and seawater and activated sludge from a wastewater treatment plant (WWTP) as inoculums. The experiments were performed in the dark at 25 °C. The main difference between the lake water and seawater inoculums was the higher conductivity, pH, and total suspended solids in the seawater compared to the lake water. The biodegradation potential was the highest for microcrystalline cellulose (MCC) followed by the different spun yarns: cotton, rayon, polyester/cotton (50:50), and finally polyester, which had the lowest biodegradation potential; see Figure 10. MCC was used in this study as reference material and showed the highest biodegradation in all tested environments with around 81% biodegradation in lake water and around 71% in seawater during 35 days. The same values for cotton were 72% and 49% in lake and seawater, respectively. The bacterial communities were also found to be promoted with MCC, cotton, and rayon in contrast to the polyester sample.^79^ The biodegradation rate, as measured by CO_2_ release, of commercial polyester and wood-base fibers in marine and aquatic assays was also compared. The wood-based cellulose fibers (lyocell) rapidly became thinner and disintegrated in 30 days, while no visual changes were observed in the polyester fibers during 200 days. Complete mineralization of lyocell fibers was confirmed in a bioreactor in 60 days and in freshwater in 90 days. The MCC reference was biodegraded even faster. Similar results were obtained when the degradation experiments were carried out in the field, aquarium, and bioreactor.^80^

## 6 Degradation during Real or Simulated Soil Burial

Understanding and confirming the degradation of polymers under soil burial is especially important for items that are expected to or have high risk of ending up in soil. This type of product could, for example, be agricultural mulch film, seed coatings, or binding yarns. Plastic mulch films have an important role in agricultural production. Nonbiodegradable mulch can fragment and pollute the soil, and it is labor intensive and difficult to collect and recycle. According to international standards, degradable mulch films should degrade to >90% CO_2_ during 24 months at 20–28 °C. The biodegradation rate under soil burial can vary greatly depending on the actual conditions, but the biodegradation rate is typically much slower compared to composting. This is explained by the generally less favorable environment with respect to, e.g., the temperature, humidity, and concentration of microorganisms. Cellulose is often used as a standard or positive control even in soil burial tests since its biodegradation time is comparatively short. Average cellulose residence times of 81–495 and 31–61 days have been reported in temperate and tropical forests soils, respectively.^81^ This clearly shows the significant effect of the type of soil and other conditions on the biodegradation rate of even readily biodegradable materials. The degradation rate will be further influenced by the type and degree of chemical modification and of course on the possible photo-oxidation caused by UV irradiation from the sun prior to the soil burial. The studies on degradation of modified cellulose material during soil burial are quite scarce.

The degradation of several materials including paper, CP, and nitrocellulose or PVDC coated CP were thoroughly investigated in four different burial sites in France with different soil and climate conditions during a 2 year period. 100% mass loss was demonstrated for paper and CP, while for the modified CP, the weight loss varied between 74% and 95% depending on the material and burial site. The difficulties in weight loss measurement because of both fragmentation and challenges in removing adhered soil and mycelium without losing some sample were clearly illustrated. A new method based on image analysis was proposed to overcome this problem to enable better reproducibility.^82^ The weight loss of plastic films consisting of CMC/gelatin/agar was followed during laboratory soil burial experiments. Quite fast weight loss was observed, reaching 13–29% during the first 3 days depending on the composition and increasing then to 92–96% after 7 days.^83^ Degradation of cellulose thiocarbamate and cellulose acetate thiocarbamate was also investigated and compared with biodegradation of cellulose under anaerobic soil conditions. The compounds having carbamate and thiocarbamate groups grafted on their main chains were generally less susceptible to biodegradation. The degradation of the samples aged under aerobic solid conditions was only visually evaluated.^84^

CA produced from rice straw remained almost unchanged during first 30 days of soil burial but then degraded to be nonvisible during 105 days and had accelerated mass loss after 90 days. The degradation was followed only by weight loss. During same time period, PET remained almost unaffected and Mater-Bi films still remained intact.^85^ The weight loss of CA/γ-poly(glutamic acid) 70/30 electrospun membranes proceeded rapidly during first 6 days of soil burial, reaching approximately 30%, which corresponds well with the weight % of γ-poly(glutamic acid) in the blends. Further weight loss proceeded slowly reaching approximately 50% after 30 days, indicating that CA was significantly more persistent against degradation.^86^ Functionalization of cellulose fibers with nanopowder of elemental silver reduced the biodegradation rate of the fibers during soil burial experiments.^87^ The evaluation of biodegradability of nanocellulose films under soil burial in the laboratory in three different soil types showed the importance of moisture content. The films placed in soil with the highest moisture content exhibited fastest weight loss, and the films were no longer observable after 14–35 days, while this could take up to 40 days in soil with lower moisture content. However, CO_2_ formation was again not measured, so the weight loss could also partially be due to dissolution of the films.^88^

## 7 Degradation during Real or Simulated Composting

Composting is an environmentally friendly biological and chemical transformation process for converting organic waste into CO_2_, water, and value-added products, such as humic substances, that can improve soil quality. Compostable materials should leave no visible or toxic residues. Compost presents a favorable degradation environment with ideally high humidity, elevated temperatures (50–60 °C), and high concentrations of microorganisms (∼10^9^ per mL). Several international standards exist for testing and confirming the compostability of polymer materials. Generally, the requirement is >90% degradation during 6 months. While pure cellulose readily degrades in good compost (97% degradation within 47 days),^89^ lignocellulose (e.g., agricultural residues and wood) does not degrade as readily. A recent review summarizes the mechanisms and different pretreatments that can be utilized for accelerating the biodegradation of lignocellulosic biomass.^90^ The compostability of different cellulose modifications to be used in material applications has also been evaluated in several papers through simulated composting experiments in the laboratory or by composting in real-composting facilities of different types.

The degree of biodegradation during composting can be measured by the release of CO_2_. A few studies can be found where the biodegradation of modified cellulose materials during composting was followed by CO_2_ release. CA films were exposed to simulated thermophilic compost environments at approximately 53 °C. A reduction of the DS from 1.7 to 1.3 and from 2.5 to 2.2 was observed after 12 days. No significant changes were observed for the films aged under the corresponding abiotic conditions. The same samples were also aged in respirometry to follow the CO_2_ evolution. Interestingly, the lag phase before CO_2_ production increased from 10 to 25 days when the DS increased from 1.7 to 2.5. Furthermore, 72% and 76% of theoretical CO_2_ was recovered after 24 and 60 days for the CA with a DS of 1.7 and 2.5, respectively.^91^ This study clearly demonstrated that even the biodegradation rate during composting is significantly influenced by the DS. However, the DS was shown to decrease during the process, which should facilitate subsequent biodegradation after the lag phase. The same authors in a further study evaluated the effect of compost mixtures and humidity on the biodegradation process. As an example, a decrease in the humidity from 60% to 40% increased the time it took for CA (DS of 1.7) to visibly disappear from 6 to 30 days.^92^ Another study utilized microcrystalline cellulose as a reference for CA in a laboratory controlled composting test. MCC yielded a CO_2_ evolution of 67% of the theoretical value in 55 days of incubation. In contrast, the CA materials with a DS of 1.5, 2.5, and 3 yielded a CO_2_ evolution of 50%, 45%, and 9%, respectively, for the same composting period.^93^

**Figure 11:**
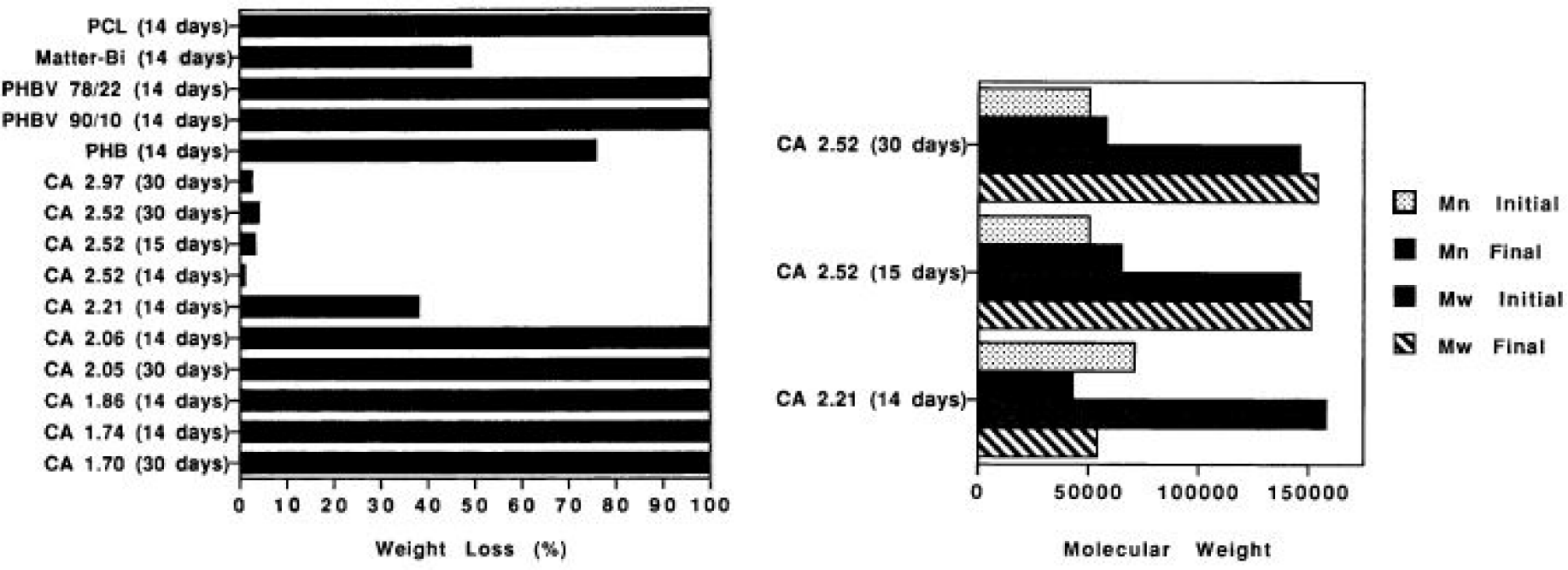
Effect of the DS on compostability of CA as measured by weight loss and molecular weight changes. Reprinted with permission from ref (94). Copyright 1994, John Wiley and Sons.

The compostability of several CA films with DSs ranging from 1.70 to 2.97 was followed by measuring weight loss, molecular weight changes, and deacetylation. This was done in a bench-scale composting setup simulating municipal windrow composting. The cylinders were kept in a 35 °C room, but the temperature inside the cylinders could be higher depending on the compost cycle. CA with five different DSs was included, and a sharp decrease in the weight loss was observed when the DS increased to above 2 (Figure 11). The CA with a DS of 2.06 still experienced 100% weight loss during 14 days, but the weight loss decreased to approximately 37% for CA with a DS of 2.21 and further to 2–5% for CA with a DS of 2.52–2.97, even with a prolonged composting time of 30 days. Comparison of the molecular weight changes for the materials with a DS of 2.21 and 2.52 shows that a small molecular weight decrease is observed for DS 2.21 material, while both *M*_n_ and *M*_w_ slightly increased for CA with a DS of 2.52. This indicates that it could be only the molecules with shortest chain length that degraded for the DS 2.52 material. This could lead to a small increase in the average molecular weight of the remaining material. A similar influence has been shown earlier during hydrolysis of polyesters. The analysis of the DS showed a minor decrease after 30 days of composting. A longer period of time and a possibly higher temperature are thus required for significant deacetylation and initiation of biodegradation of high DS CA materials.^94^

The degradation rates of 14 different cellulose materials were compared during pilot-scale composting. The materials included regenerated cellulose, CMC, MC, CA, CP, nanocellulose, and paper, among others. Significant differences in the degradation rate were observed depending on the type of cellulose derivative. While cellulose was almost 100% degraded after 4 weeks of composting, CA (DS ∼ 2.5) did not degrade during the composting experiments. In contrast, the water-soluble derivatives of cellulose CMC-Al (CMC cross-linked with aluminum, DS of 0.7) and MC (DS ∼ 1.7) were no longer visible after 2 and 8 weeks, respectively. CC and CP were no longer detected after 6 weeks of composting and nanocellulose, after 4 weeks. This was in sharp contrast to the cellulose esters with longer substituents, cellulose octanoate and cellulose palmitate, that in agreement with the enzymatic hydrolysis test previously mentioned did not degrade at all during 12 weeks of composting. In the case of butylated hemicellulose (DS of 1), the degree of enzymatic degradation was only around 20% after 2 weeks, while the material was no longer detected after only 2 weeks of composting. However, the degradation during these composting experiments was only evaluated visually and not by CO_2_ production, so some of the reported degradation could be merely dissolution of the material and not biodegradation.^95^

**Figure 12:**
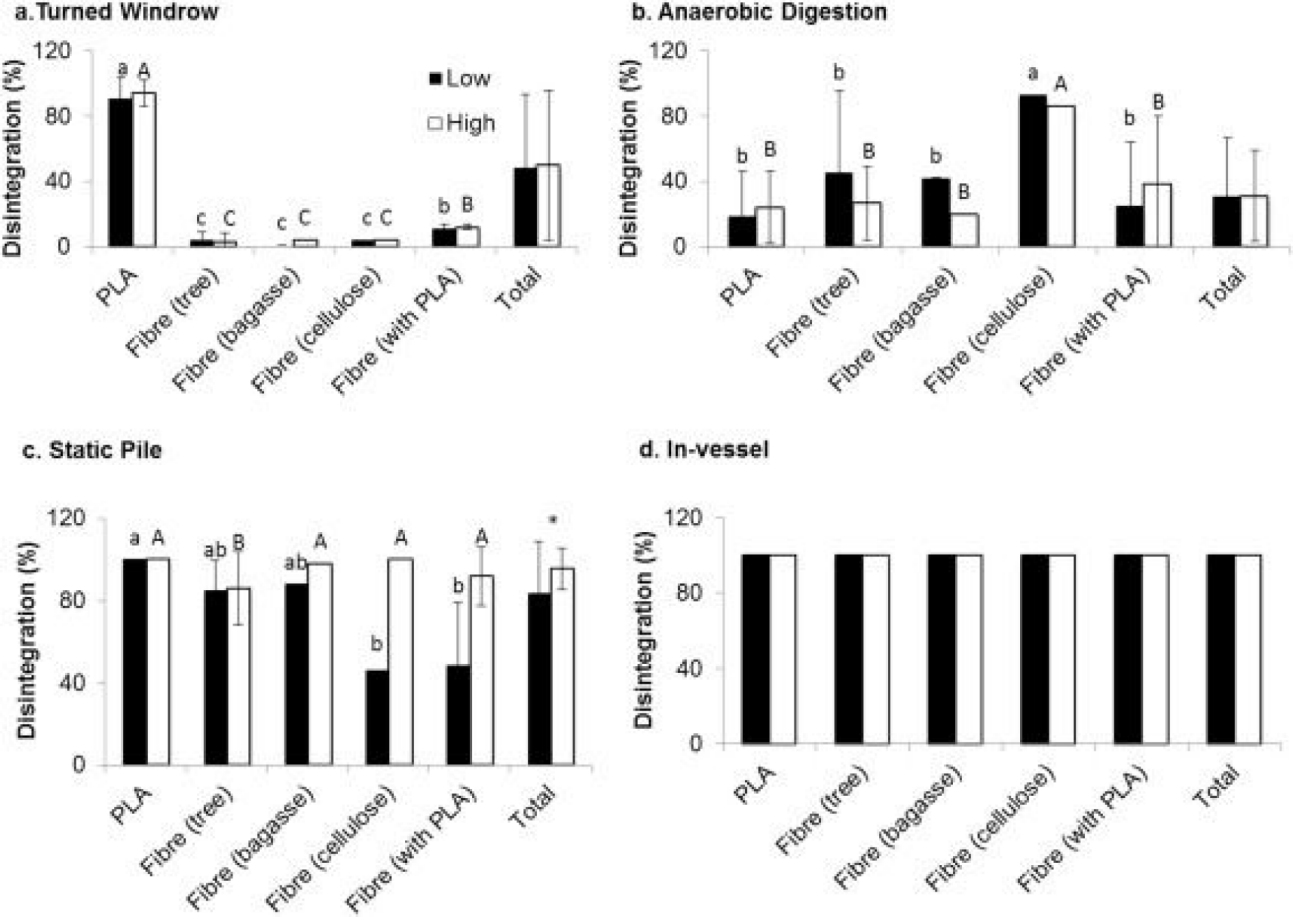
Weight loss (%) of compostable foodware and packaging products after (a) 65 days in turned window; (b) 45–49 days of anaerobic digestion; (c) 50 days in static pile; (d) 82 days of in-vessel composting. Reprinted from ref (96). *International Biodeterioration & Biodegradation*, *125*, Zhang, H.; McGill, E.; Ohep Gomez, C.; Carson, S.; Neufeld, K.; Hawthorne, I. Disintegration of compostable foodware and packaging and its effect on microbial activity and community composition in municipal composting, 157–165. Copyright 2017, with permission from Elsevier.

The disintegration rates of commercial compostable foodware packaging varied greatly depending on the type of material (PLA and different fiber-based products) and the type of composting facility, which included in-vessel, static pile, turned windrow, and anaerobic digestion (Figure 12). As an example, PLA degraded faster in turned windrows, while fiber-based products showed more degradation during anaerobic digestions. Near complete or complete disintegration for all material was achieved in static pile and in-vessel composting. However, the composting time in-vessel was also significantly longer. Again, the formation of CO_2_ was not measured, so there is no definite confirmation of the final degree of biodegradation.^96^ The addition of a small amount of PLA–PEG on bacterial cellulose films improved the water barrier properties, while a high degradation rate under soil burial at 60 °C was maintained.^97^

**Figure 13:**
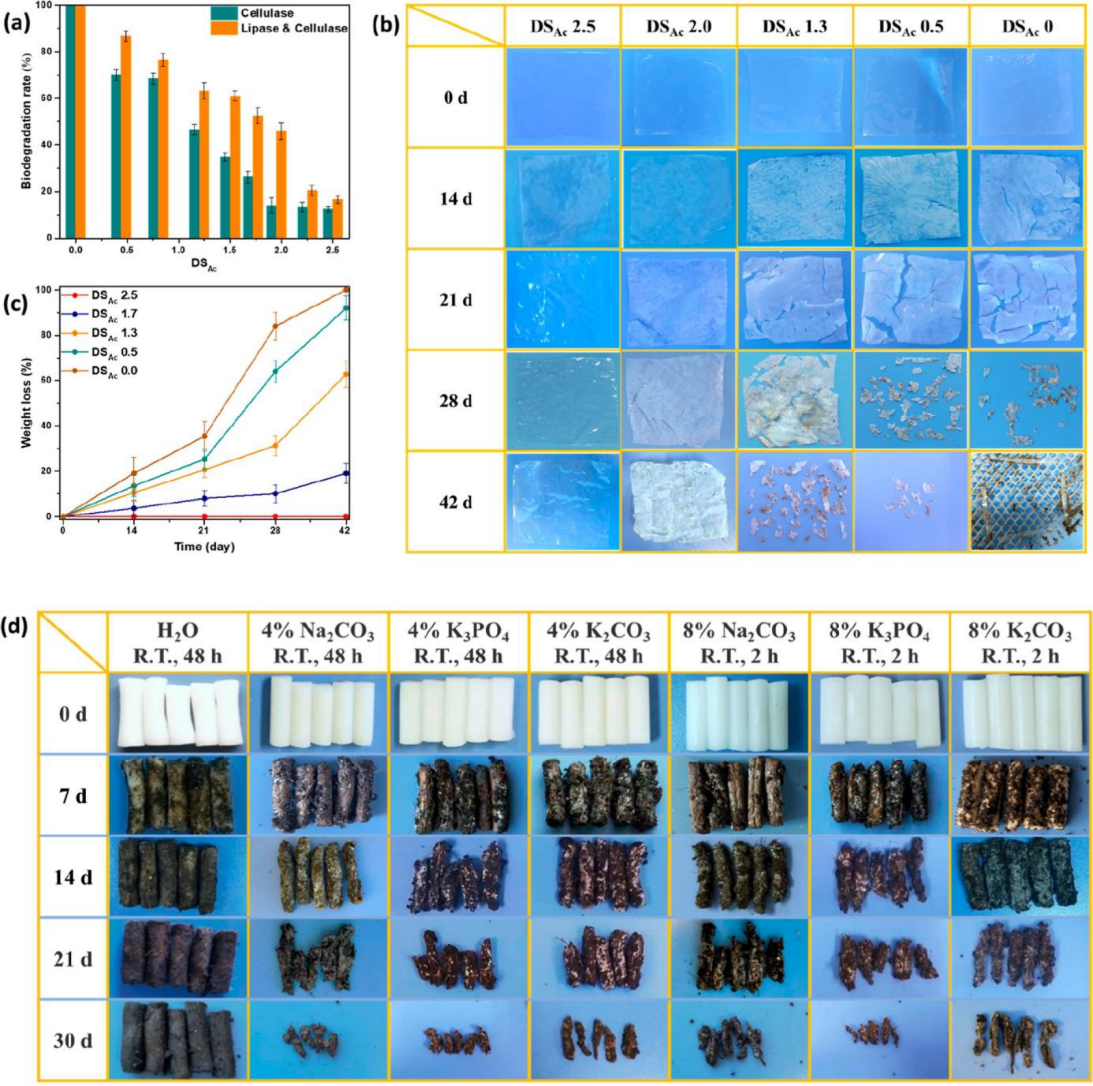
Degradation of CA with different DSs after deacetylation pretreatment. (a) Enzymatic degradation rate by cellulase and a mixture of lipase and cellulase; (b) the appearance of the films after composting up to 42 days; (c) corresponding weight loss; (d) appearance of pretreated cigarette filters after composting up to 30 days. Reprinted from ref (58). Copyright 2022 American Chemical Society.

The biodegradation of different nonirradiated or gamma or electron beam irradiated CP materials was compared in a simulated aerobic composting environment according to ISO 14855. Three different types of cellophane films, uncoated cellophane (CP), nitrocellulose-coated cellophane (CM), and PVdC-coated cellophane (CK), were studied together with cellulose. The effect of irradiation-induced sterilization on the biodegradability of the films was also assessed. Nonirradiated uncoated cellophane (CoCP), nonirradiated nitrocellulose-coated cellophane (CoCM), and nonirradiated PVdC-coated cellophane (CoCK) demonstrated around 71%, 55%, and 63% mineralization, respectively, after 141 days. The same value for the positive control cellulose was 87%. When the cellophane films had been gamma or electron beam irradiated, the CO_2_ evolution for cellulose and uncoated cellophane became comparable during the first stages of composting. After 20 days, the biodegradation rate of irradiated CP was higher than that of the positive control cellulose. The biodegradation rate on the coated CP materials remained lower even after irradiation.^98^ The addition of biodegradable plasticizers (e.g., 20 or 30 weight % of triacetin) accelerated the biodegradation of CA (DS of 2.4) during controlled composting conditions, leading to complete biodegradation within 46 days, while the same CA without plasticizer or with phthalate plasticizer did not fulfill the requirements to be classified as compostable.^99^ Biodegradation of cross-linked and non-cross-linked MC under controlled composting conditions illustrated a decreased biodegradation rate after cross-linking with 60% lower CO_2_ emission compared to non-cross-linked MC. This could be connected to the decreased moisture absorption and increased *T*_g_ for the cross-linked materials.^100^ A facile pretreatment process was proposed to increase the biodegradation rate of CA by deacetylation. This was achieved by treatment with common salt solutions (e.g., NaHCO_3_, Na_2_CO_3_, K_2_CO_3_, and K_3_PO_4_). Depending on the treatments, the degree of acetylation was gradually decreased from 2.5 to 0, which had a significant effect and was directly correlated to the observed weight loss and fragmentation during subsequent simulated composting experiments (Figure 13).^58^

The biodegradability and compostability of CNF products was evaluated under controlled composting conditions.^101^ The samples included CNF films, concentrated CNF, and paper products containing CNF. After 65 days, the biodegradation (%) of concentrated CNF and CNF films were 76% and 100%, respectively. The biodegradation of Whatman paper and cellulose powder references was 82% and 69%, respectively, during the same time period. CNF could, thus, biodegrade even faster than paper and cellulose powder. In the same study, rapid disintegration in a pilot scale composting test was demonstrated and no acute ecotoxicity was observed after evaluation with *Vibrio fischeri*.

Compositing can significantly influence the biodegradability and compostability of materials depending on, e.g., the resulting surface roughness, hydrophilicity, and biodegradability of the added components. Several studies investigated the effects of natural fibers, CNF, or CNC on the compostability of green composites. As an example, simulated aerobic composting experiments showed that MCC, cellulose fibers, wheat, and soy straw were rapidly biodegraded. Furthermore, compositing with biodegradable polymers PLA and PCL increased the biodegradation rate compared to the biodegradation rate of the plain polymers.^102^ This influence is, however, complex and not always easily predictable. For example, the addition of microsized cellulose (MFC) significantly increased the weight loss and molecular weight loss of PLA during composting experiments, while the addition of CNC decreased both. This was explained by accelerated surface degradation in the presence of MFC, indicating that the biodegradation process started from the hydrophilic MFC.^103^ Opposite results have also been reported where the weight loss increased as a function of CNC addition to PHB/PCL blends.^104^ In accordance, the blend of bacterial cellulose with PHB also increased the biodegradation of PHB as measured by the release of CO_2_ under controlled aerobic composting conditions.^105^ The different influences could be connected to the influence of additives on the penetration of water into the materials, where hydrophilic components generally increase the water absorption and hydrolytic degradation rate and hydrophobic or barrier property improving additives have the opposite effect. This could be further influenced by the inherent biodegradability of the Bioplastic matrix where, e.g., PHB and PCL are known to biodegrade significantly faster compared to PLA. Furthermore, the ability of the nanocelluloses to function as nucleating agent could increase the degree of crystallinity, leading to lower degradation rate.^106^

## 8 Conclusions and Future Perspectives

Biodegradable materials alone cannot solve the connected waste problem, but they are one necessary puzzle piece because some polymer and fiber materials are difficult to recycle and some will end up in the environment either because of their application or due to wear of the products (e.g., textiles, coatings). In addition, while most packaging should be designed for recycling, there are special occasions like festivals, when compostable packaging could make the heterogeneous food/packaging waste directly compostable.^107^ Here, cellulose derived materials are of high interest.

Weight loss and breakdown of plastics to fragments and smaller molecules are commonly observed during the degradation process. Degradation of nonbiodegradable plastics may lead to persistent microplastics, while fragments and microplastics produced from inherently biodegradable materials are more likely further biodegraded and ultimately mineralized. Following the weight loss and changes in materials, physical, chemical, and mechanical properties and the release of degradation intermediates give an important mechanistic understanding concerning the degradation process, but it is not enough to claim the material is completely biodegradable and environmentally benign. Complete conversion to CO_2_ or CO_2_ and CH_4_ should be proven to claim ultimate biodegradability. The fate and transport of biodegradable plastics, such as different cellulose derivatives, their incorporated additives, intermediate degradation products, and formed microplastics, as well as their effect on soil and marine ecosystems should also be further investigated.^18,108^

There is a need to better understand the degradation processes and correlations between controlled and reproducible laboratory tests and less-defined open environment degradation. Intrinsic degradability demonstrated under favorable laboratory conditions might not always translate to real degradation in real environment due to, e.g., the absence of suitable microorganisms or low temperature and humidity. A better understanding of these correlations enables correct conclusions from laboratory testing under simulated or accelerated conditions including how these results can be extrapolated to degradation in real natural environments. The materials to be investigated should be well-characterized and have known compositions to understand the structure–degradability relationships and what is facilitating or preventing the degradation process.

The degradation of commercial cellulose derivatives has been investigated under different laboratory conditions as well as in real natural, agro-industrial and man-made environments. It is clear that the types of modification and the degree of substitution in combination with the actual degradation environment are important factors influencing the susceptibility to degradation and degradation rate. For CA, a sharp decrease in biodegradation rate is generally observed when the DS approaches 2 and above. Unfortunately, at the same time, CA with DS > 2 is generally required for thermoplastic properties and good thermal processability. An increase in the length of the ester group rapidly decreases the biodegradation rate observed for materials with similar DSs. For cellulose ethers, like CMC, the limit of ready biodegradability is typically already observed at DS > 1. Still, the literature is fragmented, and there is need for new systematic studies that investigate the correlation between chemical modification, physicomechanical properties, processability, and degradation under the influence of different environmental parameters. This need is further catalyzed by the rapid development of the field with many new cellulose-based materials designed with unknown degradation behaviors. These investigations could beneficially be combined with the utilization of new characterization and computational tools like machine learning. A recent study, for example, constructed a database consisting of a degradation experiment from the literature and different material parameters such as chemical structure and physical properties (e.g., hydrophobicity, glass transition, crystallinity, density, and molecular weight). This database and machine learning were utilized to rank and predict the influence of different chemical and physical structures on the degradation potential of marine debris.^109^ However, no cellulose-based materials were included.

When one designs new cellulose-based materials, the targeted end-of-life environment should be taken as one of the design principals. Depending on the applications, materials should be designed for material recycling or “biological recycling” in specific predetermined environments. Some concepts from the design of synthetic polymers to the degradation in different environments by, e.g., the introduction of weak linkages in the backbone^110,111^ might be difficult to apply when working with high molar mass biopolymers. Other modifications such as the utilization of dynamic covalent chemistry and covalent adaptable networks could be important tools for turning cellulose materials and fiber composites into high performing circular materials.^112^ The embedment of different functionalities and labile or reversible chemical bonds or green additives, including photocatalytic compounds or enzymes, could be utilized to trigger the disassembly of the materials when subjected to UV irradiation, heat, moisture, or pH.^113,114^
